# The Beat

**Published:** 2007-09

## EPA Testing Program Raises Concerns

In June 2007 the EPA released for public comment a draft list of 73 chemicals to be screened for their potential as endocrine disruptors. With a comment period extending to 17 September 2007, a number of scientists and health advocates already have weighed in with their concerns about the testing program as a whole. They are alarmed by what they see as undue influence by the chemical industry on the development of the testing process, a claim rebutted by the EPA, which retorts that the process has been open and transparent. An article in the 5 July 2007 *Dallas Morning News* lists, among other issues, concerns that the tests as designed may miss chemicals that could cause health effects, that testing is not required to include prenatal exposures, and that incorrect dosage ranges will be tested, possibly missing certain effects.

## Lead Swabs Miss the Mark

Lead can cause significant health effects in children at blood concentrations lower than 10 μg/dL. One significant source of lead in homes is dust created by crumbling lead-based paint. To detect lead in household dust, homeowners, landlords, and health advocacy groups often use LeadCheck® Swabs as a cheap and quick alternative to more expensive and time-consuming EPA-approved dust wipes, which must be analyzed by a laboratory to confirm results. But in the June 2007 issue of *Environmental Research*, University of Rochester researchers testing the swabs under typical field conditions found that more than 60% of dust samples deemed to have passed muster actually contained hazardous concentrations of lead. The authors write that household dirt may affect the swabs’ sensitivity and suggest that guidance for the use of such kits “should clearly explain the risks of false negatives and lay out appropriate follow-up actions when negative results are achieved.”

## Have Another Cup

**Figure:**
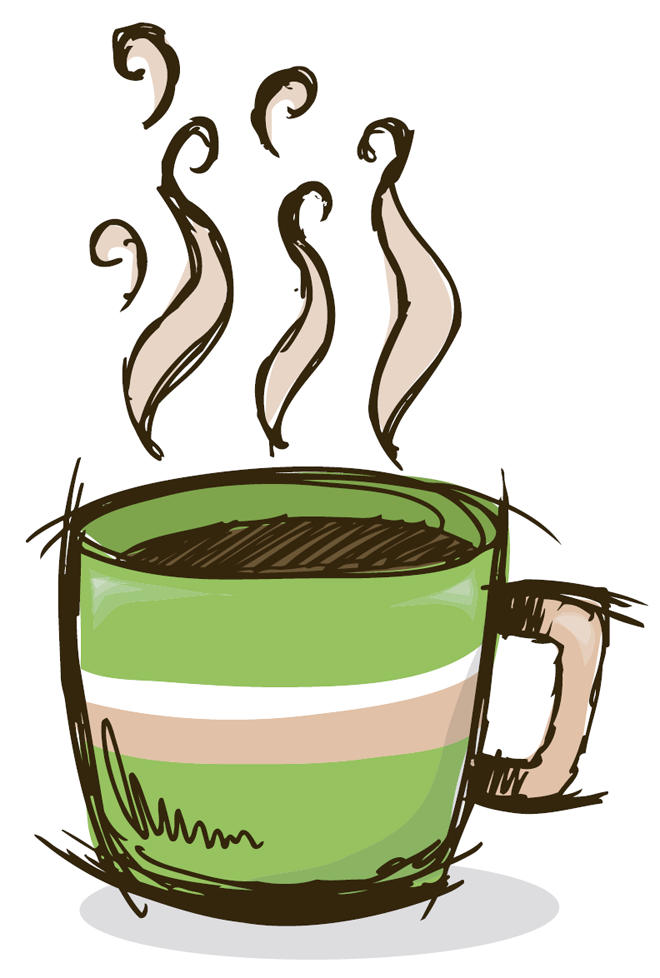


Good news for coffee lovers! Researchers from Sweden’s Karolinska Institute report in the May 2007 issue of *Gastroenterology* the results of a meta-analysis of eleven studies over the past two decades. Their findings show an inverse relationship in all the studies between the amount of coffee consumed and risk of liver cancer. The association was statistically significant in six of the studies. More specifically, a 43% reduced risk of liver cancer was observed with an increase in consumption of two cups of coffee per day. The researchers note that the effect could be due to the presence in coffee of large amounts of chlorogenic acids—antioxidants that reduce oxidative stress and have inhibited liver cancer in animal studies.

## Rethinking Sources of Perchlorate

Although best known as a pollutant linked to its use in rocket fuel, perchlorate also made its way into U.S. agricultural soils from Chilean nitrate fertilizers that came into use in the early 1900s. But the chemical, which disrupts thyroid function, also occurs naturally in the environment, notably in South America’s Atacama Desert. Scientists now know that perchlorate hot spots are not limited to known contamination or natural sources. In an *Environmental Science & Technology* article published online 6 June 2007, Texas Tech and USGS researchers report that a substantial reservoir of natural perchlorate exists in unsaturated zones (between the land surface and the water table) of the U.S. Southwest, and that this reservoir is large enough to affect drinking water, groundwater, and crops when irrigation or precipitation flushes the chemical from the soil. The authors write that this may help explain increasing reports of perchlorate in dry-region agricultural products.

## New Guidance for Supplements

On 22 June 2007, the FDA released its long-awaited final rule on dietary supplement good manufacturing practices. The move was hailed by many in the industry, although some policy makers and consumer advocates believe the regulations don’t go far enough to ensure the safety of supplements, now a $22 billion industry. The rule focuses on accurate representation of ingredient content on labels, production process quality, and elimination of impurities and contaminants from products. The FDA may also inspect manufacturing plants for compliance. Manufacturers are charged with keeping records documenting that ingredients have undergone appropriate testing. The rule also applies to supplements produced outside the United States. However, according to the Consumers Union, the rule “still does nothing to ensure that supplements are safe or effective before they go on the market.”

## No Link between Hair Relaxers and Breast Cancer

**Figure:**
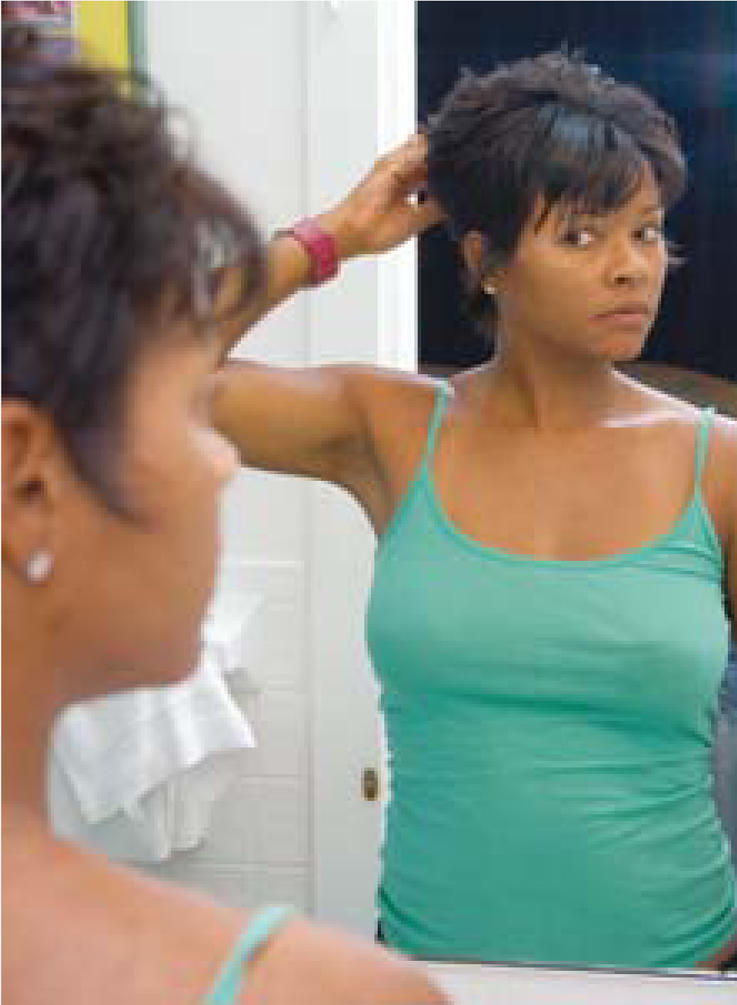


Hair relaxers are used by millions of black women, often over many years. Researchers from Boston University and Howard University studied the use of these products to determine if they may play a part in why breast cancer incidence is higher among young black women than young white women. An article in the May 2007 issue of *Cancer Epidemiology, Biomarkers & Prevention* reports no increase in breast cancer risk among women who use hair relaxers, even those who had used them frequently and for long periods of time. The study is the first to assess hair relaxers as a potential contributor to cancer.

